# I’ve looked at gut from both sides now: Gastrointestinal tract involvement in the pathogenesis of SARS-CoV-2 and HIV/SIV infections

**DOI:** 10.3389/fimmu.2022.899559

**Published:** 2022-08-12

**Authors:** Ivona Pandrea, Kelsie Brooks, Rahul P. Desai, Minali Tare, Jason M. Brenchley, Cristian Apetrei

**Affiliations:** ^1^ Department of Pathology, School of Medicine, University of Pittsburgh, Pittsburgh, PA, United States; ^2^ Department of Infectious Diseases and Microbiology, Graduate School of Public Health, University of Pittsburgh, Pittsburgh, PA, United States; ^3^ Barrier Immunity Section, Laboratory of Viral Diseases, Division of Intramural Research, National Institute of Allergy and Infectious Diseases, National Institutes of Health, Bethesda, MD, United States; ^4^ Division of Infectious Diseases, Department of Medicine, School of Medicine, University of Pittsburgh, Pittsburgh, PA, United States

**Keywords:** HIV - human immunodeficiency virus, SIV, SARS-CoV-2, AIDS - acquired immunodeficiency syndrome, COVID - 19, inflammation, microbial translocation, barrier integrity

## Abstract

The lumen of the gastrointestinal (GI) tract contains an incredibly diverse and extensive collection of microorganisms that can directly stimulate the immune system. There are significant data to demonstrate that the spatial localization of the microbiome can impact viral disease pathogenesis. Here we discuss recent studies that have investigated causes and consequences of GI tract pathologies in HIV, SIV, and SARS-CoV-2 infections with HIV and SIV initiating GI pathology from the basal side and SARS-CoV-2 from the luminal side. Both these infections result in alterations of the intestinal barrier, leading to microbial translocation, persistent inflammation, and T-cell immune activation. GI tract damage is one of the major contributors to multisystem inflammatory syndrome in SARS-CoV-2-infected individuals and to the incomplete immune restoration in HIV-infected subjects, even in those with robust viral control with antiretroviral therapy. While the causes of GI tract pathologies differ between these virus families, therapeutic interventions to reduce microbial translocation-induced inflammation and improve the integrity of the GI tract may improve the prognoses of infected individuals.

## Introduction

**Figure 1:**
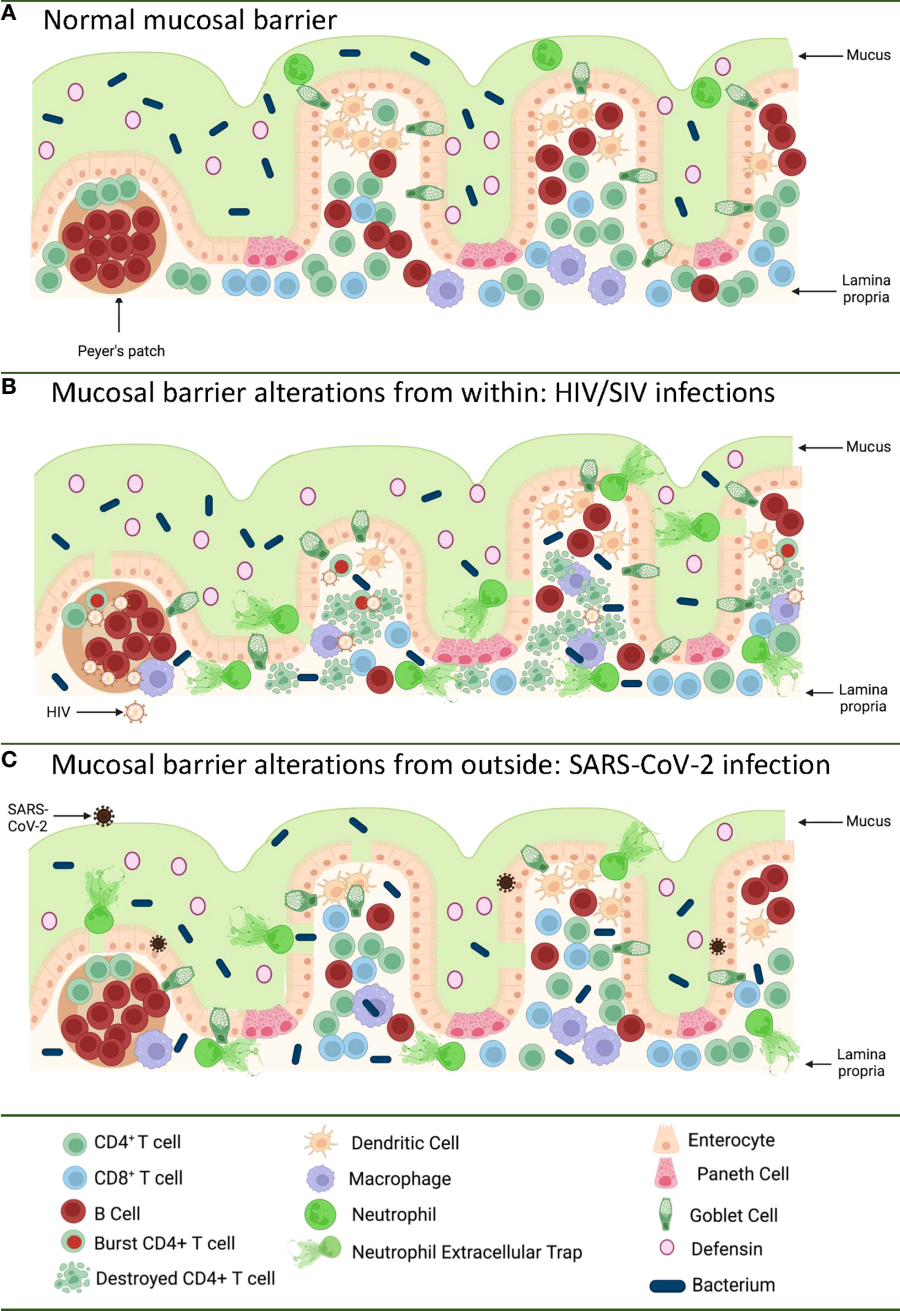
Pathways of the gastrointestinal tract damage in HIV/SIV and SARS-CoV-2 infections. **(A)** Normal GI tract is a continuous barrier which protects the internal milieu by the damage of an enormous microbiota existent in the GI lumen. This barrier is multistratified, being represented by mucus, a continuous intestinal epithelium, and immune effectors that capture translocated microbes. **(B)** While HIV/SIV penetrates the body at mucosal sites, GI infection occurs through systemic seeding. CD4^+^ T cell destruction and the inflammatory responses contribute to the destruction of the mucosal barrier from within, leading to the translocation of the intestinal flora in the lamina propria and then systemically; **(C)** SARS-CoV-2 infection of the enterocytes (that express high levels of the ACE-2 and TMPRSS-2 receptor) produce direct epithelial destructions also leading to translocation of the intestinal microbiota to the lamina propria and then systemically. Mucosal damage is both a major determinant of long COVID, as well as of an incomplete immune recovery even in HIV-infected individuals receiving suppressive antiretroviral therapy. Was created with BioRender.com.

Differently from Joni Mitchell, the Canadian-American singer-songwriter and painter who doesn’t know love at all (in spite of looking at it from both sides), we know gastrointestinal (GI) tract tissue as an immune organ very well. It contains about 80% of the total leukocytes in the body (1), and most of the human microbiota (2–4) (**Figure 1A**). The GI tract is constantly exposed to foreign antigens from food and this exposure is critical for normal development of the mucosal immune system and immune tolerance (5–8).

The GI tract has the largest surface area exposed to the environment and the intestinal epithelia confers protection against toxic substances from food and microbes, both those normally present in the human microbiome, as well as those carried with food and water. The intestinal mucosal barrier is multilayered, with intestinal mucus, commensal bacteria, GI tract epithelium and the lamina propria immune system all contributing to host defense (9, 10) (**Figure 1A**). Protection is not limited to a physical barrier, but includes numerous active cell populations that exert secretory functions: goblet cells which produce mucus glycoproteins (11, 12); Paneth cells which produce antimicrobials that have the ability to specifically lyse bacteria (13); and B cells from the lamina propria which produce immunoglobulins (IgA) that capture bacteria that arriving to this gut level, preventing their successful translocation (14) (**Figure 1A**).

Breaches of the mucosal integrity of the GI tract are central to the pathogenesis of multiple chronic metabolic, autoimmune, and aging-related diseases (9, 10). Multiple infections can alter the integrity of the mucosal barrier including (15): human and simian immunodeficiency viruses (16–21); influenza virus infection (22); dengue (23); hepatitis B virus infection (24); hepatitis C virus infection (25); and SARS-CoV-2 (26, 27).

Furthermore, the quality of our intestinal microbiome is driving our overall morbidity (28–32). An inflammatory flora, such as the one associated with a Western diet (i.e. rich in saturated fats and sugars) drives a state of chronic inflammation, which triggers multiple systemic diseases and is roughly responsible for more than 50% of the deaths on the planet (33). Replacement with a healthy diet (i.e., Mediterranean diet rich in fiber, minerals and vitamins, and Omega 3) can alter the microbiome in as little as 3 weeks and change its phenotype to an anti-inflammatory one (34–42).

The interplay of the GI tract immune system and pathogens which disrupt this complex mucosal barrier is critically important in understanding pathogenesis, and providing targets for reducing damage. We will explore the well-studied impacts of HIV and SIV on the GI tract in addition to the parallels and distinctions that can be made in a recently emerged pandemic virus, SARS-CoV-2, and its corresponding disease, COVID-19.

## Breaching the barrier from within: Mucosal pathogenesis of HIV and SIV infection

Even since the discovery of HIV, the involvement of the GI tract in the pathogenesis of AIDS was suggested by the high frequency of the gut dysfunction and wasting disease (43). Yet, the paradigm of HIV infection as a mucosal disease emerged only after the detailed characterization of the interactions between HIV and SIVs and their CD4-expressing target cells. It was reported that only the CD4^+^ T cell subsets that expressed high levels of CCR5 (i.e. central memory cells, transitional memory cells, and effector memory cells) are preferentially targeted by HIV and SIV (44–47) and that the main reservoir is represented by the central memory cells (48). From a functional perspective, Th-17 CD4^+^ T cells contribute to the maintenance of the gut integrity and are preferentially lost during progressive HIV and SIV infections (49–51). As such, since the vast majority of the effector memory cells are located at mucosal surfaces, numerous studies have shown that the first major immunologic injury inflicted by HIV/SIV to the immune system is the massive depletion of mucosal CD4^+^ T cells (>95%) that occurs at the mucosal sites within three weeks from infection (52–54). As memory CD4^+^ T cells are the preferential targets of HIV infection, their depletion is more prominent at the effector sites, such as the lamina propria, compared to inductive sites (i.e. the Peyer patches) which contain naïve CD4^+^ T cells (55). CD4^+^ T cell depletion within effector sites persists throughout chronic infection, irrespective of the virological and clinical outcome (56). Furthermore, differently from the circulating CD4^+^ T cells, which can be rapidly restored to preinfection levels after administration of combination antiretroviral therapy (cART), mucosal CD4^+^ T cell restoration is slow and incomplete (35-50% from the baseline levels) (56–58).

The severe immunologic insult produced following the interactions between HIV/SIV and their target cells within the GI tract trigger key pathogenic features of chronic SIV/HIV infection that drive disease progression (**Figure 1B**). Indeed, Th17 cells contribute to the maintenance of GI tract immunity through induction of mucins, claudins, and defensins, which are key components of the mucosal junctions and have antimicrobial activities; therefore, loss of Th17 cells directly compromises mucosal integrity (59). Their loss results in reduced levels of IL-17 and IL-22, which promote the recruitment of neutrophils and myeloid cells at the effector sites of the mucosa and lead to growth of epithelial cells (59–61). Alteration of the Th17/Treg ratio is associated with increased indoleamine-2,3-dioxygenase (IDO) expression by antigen-presenting cells (62–65). IDO is involved in the tryptophan metabolization (64), and IDO metabolites directly inhibit Th17 cell differentiation (66). IDO increases are also associated with decreased frequencies of CD103 antigen-presenting cells, which can induce Th17 cells (67). Altogether these features, which are specifically associated with pathogenic SIV infection and absent during the SIV infection of natural NHP hosts (in which Th17 cells are preserved) (49, 51), point to a vicious circle that leads to a continuous depletion of the Th17 population, the consequence of which is the occurrence and intensification of the mucosal damage during HIV/SIV infections.

The impact of HIV/SIV infection on the innate immune cell populations at the mucosal sites has also been extensively investigated. Progressive HIV and SIV infections lead to a reduction of both plasmacytoid dendritic cells (pDCs) and myeloid dendritic cells (mDCs) in both the peripheral blood and spleen, and alter their homing to the gut (68). Progressive infection leads to their excessive activation, leading to increased turnover in tissues (68). Similar profiles of increased apoptosis and an altered functional profile upon HIV/SIV infections are observed for the gut-resident innate lymphocyte type III cells (69–71). As a result, instead of facilitating control of the virus through recruitment to the mucosal sites, the innate immune cells produce excess of cytokines; meanwhile their high mortality triggers release of more inflammatory cytokines by the surrounding cells, further enhancing mucosal inflammation and epithelial cell activation (72). Interestingly, mDC and macrophage recruitment to the mucosal sites also occurs during the nonprogressive SIV infections of the natural hosts or controller rhesus macaques (68). This process is, however, only transient, is not associated with excessive production of inflammatory cytokines, and does not result in their excessive death, strongly suggesting that the fate of the immune cell subsets and their functions in the GI tract is driven by the local environment (73). As such, the current view is that, being programmed to fight against the infections, the innate cells migrate to the gut in progressive, as well as in nonprogressive and controlled SIV infections. Yet, the innate cells become hyperactivated only in the pathogenic infections, due to their mucosal environment, which is altered by both the virus and translocated microbial products, and thus further fuel the inflammation, deepen the damage of the mucosal barrier, and contribute to the negative outcome of HIV/SIV infection (74, 75) (**Figure 1B**).

The HIV/SIV-associated immunological alterations at the mucosal sites result in structural and functional pathologies of the GI tract. Virus replication, inflammation and immune activation together with bystander apoptosis of the epithelial cells throughout the GI tract result in enterocyte loss and alterations of mucosal integrity (16). Progressive HIV and SIV infections trigger enterocyte loss through multiple mechanisms: (i) the virus itself can decrease glucose uptake by enterocytes through a Tat-mediated microtubule disruption or through GP120 binding to GPR15 on epithelial cells (76, 77); (ii) increased enterocyte apoptosis occurs through bystander effects, similar to other colitis (i.e. celiac disease) (78); (iii) excessive production of inflammatory cytokines (i.e., tumor necrosis factor-TNFα by innate and adaptive immune cells from the lamina propria) at the mucosal sites lead to increased apoptosis of the epithelial cells and perturbations of the tight junction epithelial barrier (79); and (iv) loss of IL22-producing innate lymphoid cells and Th17 cells leads to decreased proliferation of enterocytes (59, 61). Loss of epithelial GI tract integrity through any of these mechanisms in progressive HIV/SIV infection is associated with inflammation (80–83).

Enterocyte loss and subsequent intestinal alterations are associated with: (i) low levels of serum citrulline (a protein that is produced by the enterocytes); (ii) decreased ratio of the villous height/crypt depth (i.e., atrophy) (84); (iii) hyperproliferation of the crypt stem cells (resulting in malabsorption) (85); (iv) Increased plasma levels of the biomarkers of enterocyte damage, i.e., intestinal fatty acid binding protein (I-FABP) (86); (v) abnormal enterocyte differentiation through alterations of the sodium glucose transport and of the concentrations of intraepithelial calcium (87–89). GI tract dysfunction occurs as early as 14 days during progressive HIV/SIV infections and is associated with colitis, diarrhea, and malabsorption (43, 90).

These pathologies are specific to pathogenic SIV infections in macaques and absent during nonpathogenic SIV infections of the African nonhuman primates that are natural hosts of SIV (91–93). In these species, the mucosal lesions characteristic to pathogenic SIV infections do not occur during either the acute or chronic stages of infection (84, 94) due to an exquisite ability to maintain gut health throughout the SIV infection (94, 95).

## Breaching the barrier from outside: Mucosal pathogenesis of SARS-CoV-2 infection

SARS-CoV-2 is the etiological agent of COVID-19, a respiratory disease characterized by severe pneumonia and a plethora of symptoms suggestive of viral pneumonia: cough and sputum production, sore throat, shortness of breath, fever, myalgia, and fatigue (96–99). However, despite SARS-CoV-2 infection’s main clinical presentation as a respiratory tract infection, it may also cause symptoms associated with multiple organs, including the GI tract (diarrhea, anorexia, nausea, vomiting, and abdominal pain), liver (abnormal enzymes levels), pancreas (pancreatitis), kidney (proteinuria and hematuria, abnormal creatinine levels), brain (strokes, seizures, confusion, and brain inflammation), heart and blood vessels (elevations of cardiac injury biomarkers, palmus, chest distress, cardiac inflammation and injury, arrhythmias, and blood clots), eyes (conjunctivitis, membrane inflammation), anosmia (loss of smell), and ageusia (loss of sense of taste) (100–124).

To enter target cells, SARS-CoV-2 engages angiotensin-converting enzyme 2 (ACE2) as the entry receptor and serine protease TMPRSS2 for the Spike (S) protein priming (125, 126). Use of ACE2 is shared with SARS (127), but not with MERS, which uses a different receptor, DPP4 (128). ACE2 is widely distributed in the body, being identified in up to 72 tissues (129), and SARS-CoV-2 infection is likewise highly disseminated (130). The ACE2 protein is expressed in enterocytes, renal tubules, gallbladder, cardiomyocytes, male reproductive cells, placental trophoblasts, ductal cells, eyes, and vasculature (131). Notably, limited ACE2 expression is observed in the respiratory system both on the protein and mRNA level (132). However, a relatively limited number of cells express high levels of both ACE2 and TMPRSS2: Type II pneumocytes, nasal secretory cells, and absorptive enterocytes (131).

ACE2 expression in the human respiratory tract is highly heterogenous, being highest within regions of the sinonasal cavity (in the nasal ciliated cells) and pulmonary alveoli; these are the sites of viral transmission and severe disease development, respectively (133–137). In the lung parenchyma, ACE2 is expressed on the apical surface of a small subset of alveolar type II cells, where it was colocalized with TMPRSS2 (133–137). Interestingly, ACE2 protein expression is not reported to be lower in children, who have a lower incidence of severe COVID-19, in some studies (133); however, other investigations have described lower levels of the protein transcript in children’s airways (138).

ACE2 expression is increased in physiologic and pathologic circumstances: smoking is correlated with increased expression of the *ACE2* gene in the upper airway, but lower expression in certain lung cells (139). As such, smokers are 14 times more likely to develop a severe form of the disease (140). Interferon and influenza increase *ACE2* in human nasal epithelia and lung tissue (131). Some ACE2 inhibitors (i.e. lisinopril) have the ability to raise tissue levels of ACE2 in mice (141), while other studies did not find an increase of ACE2 expression in people treated with ACE2 inhibitors (137). Severe COVID-19, which is associated with high levels of inflammatory cytokines (IL-1β and type I and type III interferons), upregulates ACE2 expression, which has the potential to increase target cell availability and, thus, viral replication (131, 134, 139, 142). Yet, the impact on the variations of ACE2 expression on disease severity it is not known, and recently, it was reported that interferon-stimulated expression of ACE2 yields a truncated isoform that cannot bind SARS-CoV-2 (143).

Different clinical conditions were also reported to modulate ACE2 expression: hypertension, hyperlipidaemia, diabetes, chronic pulmonary diseases, and aging (134) (144). All these conditions are also risk factors for more severe clinical expression of COVID-19 (145–157). Note, however, that these data regarding ACE2 are highly debated and, to date, no comorbidity has been unambiguously associated with ACE2 expression level (144).

Several molecules were reported as alternative receptors for SARS-CoV-2, such as the C-type lectins DC-SIGN and L-SIGN (158–160), and TIM1 and AXL (161, 162). However, lectins and phosphatidylserine are not classical receptors for the virus: they are nonspecific and do not function efficiently in binding SARS-CoV-2 in the absence of ACE2 (163). Therefore, it was proposed that a more correct term for these molecules would be that of ‘attachment factors’ (144). CD147 is a transmembrane glycoprotein expressed ubiquitously in epithelial and immune cells, that was proposed as a receptor for SARS-CoV-2, yet its role as a viral receptor is downplayed by the observation that CD147 cannot bind to the S protein (164–166). Neuropilin 1 (NRP1) was also reported to be a host factor for SARS-CoV-2 (167, 168). NRP1 is expressed in olfactory and respiratory epithelial cells (167), yet its expression is low in the SARS-CoV-2 target cells (ciliated cells) and high in the goblet cells, which are not susceptible to SARS-CoV-2 (134, 169). B0AT1 is a virus cofactor that is expressed in the GI tract and kidney, but not in the lung; B0AT1 expression in the small intestine depends on interaction with ACE2 (170). Additional human genes are important for SARS-CoV-2 infection of lung epithelial cells: the GTPase encoded by *RAB7A* is critical for endocytosis, and *CTSL* encoding cathepsin L contributes to SARS-CoV-2 spike cleavage; yet more genes support other viral life cycle stages (171). Integrins were also reported to mediate cell entry of SARS-CoV-2 (172, 173), although other studies did not confirm these observations (174). Reduction of human *ACE2* in the epithelia of K18 transgenic mice in concert with increased *CTSL* did not alter the pathogenesis of SARS-CoV-2 (175), further suggesting the importance of the interplay between host factors at mucosal sites for successful viral entry and propagation.

Enterocytes express ACE2 and support viral replication that is enhanced by TMPRSS2 and 4 (176, 177), and SARS-CoV-2 virions have been directly visualized in the GI tracts of COVID-19 patients (178). SARS-CoV-2 infection rapidly induces activated CD8^+^ T cell infiltration to the intestinal epithelium (179) and increased effector CD4^+^ and CD8^+^ T cells in the lamina propria (180). This is in spite of a lack of gross pathological changes in histological findings on endoscopy in the same patients (179, 180), though others have reported abnormalities such as crypt hyperplasia with necrotic cell debris in the absence of inflammation following a positive SARS-CoV-2 test (181). Similar to this dichotomy, several studies have reported presence (182, 183) or absence (184) of viable virus isolation from stool, while viral RNA may be shed in feces for prolonged periods compared to respiratory tract samples (185, 186); persistence of viral antigens have also been reported in GI biopsies for approximately three months following infection while nasopharyngeal swabs were negative for SARS-CoV-2 RNA (187). Such a paucity of consensus regarding the impact of viral replication on GI inflammation and/or pathology is in stark contrast to HIV/SIV infection, in which ongoing viral replication in untreated infection is a clear determinant of mucosal and systemic inflammation, although such inflammation is reduced but not eliminated with the drastic reduction of viral replication during ART (188–190).

SARS-CoV-2 infection of an *in vitro* GI tract model demonstrates direct damage to tight junctions and upregulated proinflammatory cytokine transcripts (191) (**Figure 1C**). GI symptoms in COVID-19 have also been associated with elevated liver enzymes (192) while increased markers of inflammation such as TNFα and IL-6 have separately been associated with severe and/or fatal disease (193–195). The capability of SARS-CoV-2 to enter and replicate in GI barrier cells, with corresponding immune responses and GI symptoms, suggests GI tract damage may be a critical component of COVID-19 disease.

## Microbial translocation and its role in inflammation: Are lessons learned from HIV/SIV relevant to SARS-CoV-2?

GI tract dysfunction in progressive HIV and SIV infection leads to translocation of microbial products from the lumen. However, this phenomenon is not specific to SIV/HIV infection, and occurs in multiple clinical conditions in which mucosal epithelium is altered and gut permeability is increased (17). Microbial translocation is a key determinant of systemic inflammation, which is the most important driver of progressive HIV/SIV disease progression. The intestinal flora is large and diverse (approximately 10^14^ bacteria, fungi, protozoans, helminths, and viruses) and is composed of numerous antigens which can directly stimulate the immune system, including: peptidoglycan and lipoteichoic acid (through TLR2), lipopolysaccharide (LPS, through TLR4), flagellin (through TLR5), CpG-containing DNA (through TLR9 and other cytoplasmic sensors), and double stranded and single stranded RNAs (through TLR 7/8 and other cytoplasmic sensors) (88). Microbial translocation also includes fungal products that have relevance for immune activation and clinical outcome independently of bacterial products (196, 197). GI tract dysfunction, therefore, leads to significant inflammation with increased production of proinflammatory cytokines IL-1β, IL-6, TNFα and interferons (88).

Microbial translocation is specifically associated with progressive SIV/HIV infections and is nearly absent in African nonhuman primates that are natural hosts of SIV (198), and studies in nonhuman primates have established a direct link between microbial translocation and inflammation. Chronically SIV-infected African green monkeys (AGMs) that do not progress to AIDS maintain a healthy mucosal barrier and lack evidence of microbial translocation and systemic inflammation (91, 92, 94). However, intravenous administration of LPS, either in single dose or in prolonged administration over a three-week duration, resulted in increased levels of inflammation and coagulation markers (199). Similarly, alcohol or dextran sulphate administration to rhesus macaques increased GI tract permeability, induced microbial translocation, and resulted in increased levels of inflammation and SIV replication (200). Conversely, direct blockade of microbial translocation in progressively SIV infected Asian macaques with sevelamer, a chelator of LPS, resulted in a significant reduction of systemic inflammation and coagulation markers (201). Altogether, these studies provide direct evidence for microbial translocation as a key determinant of immune activation and associated pathologies, such as non-AIDS comorbidities, in SIV infection (202, 203).

Due to the key role of microbial translocation in the pathogenesis of HIV/SIV infection, studies have also focused on characterization of the impact of infection on the composition of the GI microbiome. Analysis of longitudinal samples from Asian macaques has shown that, while levels of enteric virus genomes increase, the bacterial microbiome is not dramatically altered (204–206). However, analyses of cross-sectional cohorts of HIV-infected and uninfected individuals routinely demonstrate the bacterial microbiomes of infected humans are altered (207–209). Recent studies have shown that one major contributor to the bacterial dysbiosis observed in HIV-infected individuals are risk factors for HIV acquisition (210, 211); when these risk factors are controlled for, significant dysbiosis is observed only in individuals with advanced HIV disease (210, 212). Moreover, while high fat diets lead to accelerated SIV disease in Asian macaques, with significantly increased inflammation (213), antibiotic-induced dysbiosis of the GI tract microbiome is insufficient to accelerate SIV disease (214).

Alteration to the GI tract virome may also play a role in disease. A significant increase in the size of the fecal virome was reported to occur in the progressive SIV infection of macaques, while no such change was detected in the nonprogressive SIV infection of AGMs (204). Furthermore, potentially pathogenic viruses, such as adenoviruses, are specifically colocalized with the areas of structural damage of the GI tract in progressively SIV-infected macaques (204). Finally, analysis of circulating microbial nucleic acids and those in tissues have demonstrated that microbes which translocate are not a representation of those present within the lumen, and the individual types of translocating organisms can be associated with prognosis (20, 205). Taken together it is clear that GI tract dysfunction, microbial translocation, and resulting inflammation play important roles in progressive HIV and SIV infections.

Alterations to the GI tract bacterial microbiome have been reported in hospitalized (215, 216) and even asymptomatic COVID-19 patients (217), though it is challenging to control for the confounding effects of diet, environment, and chronic conditions between infected and uninfected individuals to assess changes in microbial communities. K18 transgenic mice with a controlled diet and environment demonstrate dose-dependent GI tract microbiome alterations with SARS-CoV-2 infection (218), but the integrity of the intestinal barrier was not assessed. However, inflammation of the intestine itself has been implicated in SARS-CoV-2 infection, as COVID-19 patients with diarrhea demonstrated significantly higher levels of fecal calprotectin, largely produced by neutrophils and an indication of neutrophilic inflammation, which correlated with systemic IL-6 levels (219). Additionally, GI tract microbial dysbiosis and an increase in LPS-binding protein (LBP) were observed in severe COVID-19 patients over those with mild COVID-19, with LBP correlating to other inflammatory markers such as C-reactive protein (CRP) and IL-6 (220). Furthermore, bacterial proteins were found in COVID-19 patient blood plasma (220). Finally, in a comprehensive study by Giron et al., the tight junction protein zonulin was significantly elevated in COVID-19 patients with moderate or severe disease over controls, as were LBP and the product of monocyte inflammation in response to LPS, soluble CD14 (221). The levels of zonulin and LBP were correlated with a number of systemic inflammatory markers, again including IL-6 and CRP (221). Interestingly, both in Giron et al. (221) and another study from Hoel et al. investigating GI tract barrier integrity in COVID-19 patients (222), there was an increase in LBP without an increase in I-FABP indicative of enterocyte damage, suggesting that the epithelial barrier is disrupted by another means. The translocation of microbes and/or microbial products across a damaged intestinal epithelium, however, can induce systemic inflammation and contribute to the pathogenesis of SARS-CoV-2 infection (**Figure 1C**), as in HIV and SIV infection. Furthermore, intestinal dysbiosis in HIV infection was reported to be associated with low CD4^+^ T cell reconstitution, which may be relevant for COVID-19-associated lymphopenia (223).

While systemic inflammation, including that which may be induced by microbial translocation, is associated with COVID-19 mortality, there are additional mechanisms in which inflammation influences COVID-19 morbidity. Symptoms may persist or recur after primary infection, leading to the diagnosis of Post-Acute Sequalae of SARS-CoV-2 (PASC) or “long COVID-19” (https://recovercovid.org). Multisystem inflammatory syndrome can also occur in children (MIS-C) or adults (MIS-A) following COVID-19 diagnosis (https://www.cdc.gov/mis/about.html), and is manifested by severe organ system inflammation similar to Kawasaki disease that can occur in the presence or absence of viral antigen (224) and may be attributed to super-antigen-like attributes of SARS-CoV-2 spike protein (225). Notably, children and adults exhibit differential inflammatory responses during primary COVID-19, with adults demonstrating higher levels of LBP and IL-6, while healthy adult and pediatric controls were not significantly different in these markers (226). However, children with MIS had higher rates of GI symptoms than children with primary COVID-19, as well as increased zonulin, LBP, and IL-6 in the early stage of MIS-C (226, 227). Furthermore, mortality in MIS-C cases and primary severe pediatric COVID-19 is similar (228), suggesting that the high levels of inflammation in MISC-C may contribute to mortality as in adult COVID-19 cases. The impact of GI tract barrier disruption has been minimally explored in MIS-A or PASC cases, with one study reporting gut microbiome dysbiosis in adults with PASC at six months post-infection versus convalescent COVID-19 patients without PASC, who had returned to microbial communities similar to previously uninfected individuals (229). An additional study observed higher TNFα and IP-10 in the early recovery phase from primary COVID-19 in adults who would go on to experience PASC (230). Understanding the mechanisms of PASC and MIS, including GI damage, microbial translation, and resulting inflammation that may contribute to mortality, is therefore of critical importance. Insights from HIV/SIV infections that persistent immune activation and inflammation may occur with low levels or absence of viral antigen during virologically suppressive antiretroviral therapy (188–190) are the foundation upon which a more detailed knowledge of inflammation following primary COVID-19 may be built to provide prevention and treatment strategies.

## Therapeutic approaches aimed at limiting the impact of gut dysfunction on the outcome of HIV and SARS-CoV-2 infections

Although ART has dramatically improved the lifespan of individuals living with HIV, with life expectancy reaching near that of uninfected individuals (231, 232), treatment neither eliminates the virus nor all inflammation (233, 234). Therapeutics to complement ART and reduce the GI tract dysfunction and inflammation experienced from early infection on have taken many forms, from microbial products to probiotics to small peptides such as an apoA-I mimetic (235). Additionally, immunomodulatory treatments for reducing GI inflammation in inflammatory bowel diseases (IBD) have been assessed, and at least one therapy was evaluated for loss of gut barrier integrity and inflammation in a MIS-C case (227). The shared mechanisms of GI tract permeability and resulting inflammation in these infectious and chronic conditions suggest that strategies to effectively address inflammation in one condition may prove beneficial in another as well.

Gut microbiota are key regulators of GI tract immunity, and promotion of anti-inflammatory functions can be attempted in many ways, including provision of prebiotics, probiotics, and microbial metabolic products. Prebiotic therapies including bacterial energy sources such as short and long chain oligosaccharides have shown modest improvements to gut-related inflammation in HIV infected individuals, with significant reductions in CRP and IL-6 (236) or sCD14 (237). However, these studies were conducted in small numbers of individuals, and only demonstrated these effects in people not receiving cART (237) or individuals who had initiated treatment but poorly reconstituted CD4^+^ T cell counts of <350, and without significant change to gut microbiota alpha diversity (236). Polyphenol, a key component of the Amazonian fruit *Camu Camu* (CC), has also been suggested as a prebiotic candidate based on its anti-inflammatory and antioxidant properties in animal models and tobacco smokers, and is under investigation for use in HIV infected individuals (238, 239).

Directly modifying the gut microbiota through administration of microbial strains as probiotics has also been trialed in HIV patients receiving cART to mixed results: men with CD4^+^ T cell counts <350 did not experience changes in systemic inflammation with probiotics including eight bacterial strains, and may have experienced increased T cell activation (240); two additional studies with distinct single bacterial strain probiotics observed no significant changes with treatment (241, 242); a study with multi-strain bacterial probiotics has demonstrated reductions in systemic inflammatory markers (D-dimer, IL-6, CRP), but no reductions in LPS or sCD14 (243); one study has shown improved gut barrier health with lower enterocyte apoptosis in the intestine and increased Th17 cell in GALT with high-dose, multi-strain bacterial probiotics (244). Probiotic effects (or lack thereof) may be influenced by a number of factors such as the strain(s) used, dose, and duration of treatment; in the studies detailed above, gut bacterial microbiome alterations were not assessed (241) or not observed (242) in the single bacterial strain probiotic treatments, with only multi-strain treatments demonstrating changes to the microbial communities (243, 244). Attempts to alter the complex gut microbiota may therefore require complex therapeutics, and indeed combinations of pre- and probiotics (synbiotics) have been utilized. However, like their probiotic counterparts, these studies have shown mixed results, with unaltered sCD14 and CRP levels in women (245), reduction in IL-6 in ART-naïve individuals (246), and lessened gut dysfunction in ART-treated macaques (247).

Supplementation with microbial metabolic products such as short-chain fatty acids, which are produced by GI tract microbiota through fiber fermentation and promote intestinal homeostasis (248, 249), has long been sought as a means of reducing GI tract inflammation (250). A recent study utilizing sodium propionate in conjunction with cART has shown a transient increase in circulating IL-17, but consistent decline in CD4^+^ Th17 and Treg cells (251), which may not promote improved gut dysfunction.

Additional microbial therapies to promote intestinal barrier integrity warrant further investigation, however: mucosaly-associated fungi promoted IL-22 and IL-17 production in the intestine of mice, promoting barrier integrity and reducing damage during infection (252). Modulating bacterial communities to specifically reduce those associated with enhanced inflammation, rather than providing beneficial bacteria as probiotics, also may be a promising alternative approach: bacteriophage mediated delivery of CRISPR-Cas9 has successfully reduced specific bacterial strains in the intestines of mice (253).

Although most therapies for reduced inflammation induced by GI tract damage target the gut microbiota, another means of modulating dysregulated gut inflammation includes apoA-I mimetics, which bind LPS and lipids. Not only has an apoA-I mimetic peptide demonstrated reduction of HDL cholesterol *ex vivo* (254), but the molecule and another mimetic have also reduced inflammatory cytokines such as TNFα and IL-6 in the plasma of HIV-infected humanized mice (235). These peptides do not directly interact with the virus, and have already been implicated in treatment of chronic non-infectious inflammatory GI tract conditions such as inflammatory bowel disease (IBD) (255). Investigated as a complement to ART, apoA-I mimetics could be an excellent candidate for reduction of HIV or SARS-CoV-2 induced GI tract dysregulation and inflammation.

Steroids are a clear treatment for consideration to reduce inflammation, but are not components of standard therapies for individuals living with HIV. However, in an acute infection characterized by hyperinflammatory conditions such as COVID-19, the immunosuppressive effects of corticosteroids have been beneficial: in severe COVID-19 patients, moderate doses of dexamethasone administered for a short duration reduced the duration of hospitalization and mortality (256–260).

Finally, cell signaling approaches have been taken to reduce inflammation resulting from GI tract disruption. In a case of severe MIS-C, inhibiting zonulin signaling with a zonulin receptor agonist was undertaken to improve tight junctions, with tight junction loss hypothesized to lead to antigenemia and severe systemic inflammation (227). The child’s condition did improve with treatment, as evidenced by decreased CRP, D-dimer, and indeed lower SARS-CoV-2 spike protein in the blood (227). This virus-independent means of reducing GI tract disruption, which is currently approved for a clinical trial for celiac disease treatment (261), may be appropriate for HIV as well, as might anti-inflammatory treatments for other chronic immune conditions such as IBD. Although TNF antagonist and immunosuppressive thiopurine treatment was associated with risk of hospitalization or death from COVID-19, TNF antagonist treatment alone was associated with lower odds ratios of hospitalization or death (262). Treatment with anti-TNFα antibodies has proven successful at reducing inflammation in clinical trials (263) and may be a safe strategy for reducing GI tract inflammation that results from viral infection, either chronically in HIV or acutely in SARS-CoV-2; indeed anti-TNFα antibodies were successful at reducing pulmonary pathology in a case study of a COVID-19 patient (264) and in inflammation and pathology in progressive SIV infection (79). Furthermore, anti-IL-6 therapies have been investigated for HIV and SARS-CoV-2 and proposed for inflammatory gut diseases, though efficacy has been mixed for both viral infections (265–268).

In conclusion, despite the distinctions of SARS-CoV-2 and HIV infections in terms of target cells, viral persistence, and symptomatology, there are considerable parallels in the loss of gut barrier integrity and corresponding inflammation that results. These parallels suggest that therapies to address chronic HIV inflammation, as well as that of non-infectious diseases, may be appropriate for treating SARS-CoV-2. Although the infection is acute rather than chronic, MIS cases strongly suggest persistent or recrudescent damage of organ systems including the GI tract that can lead to serious and fatal inflammation. Treatment therapies to reduce GI tract damage and/or resulting inflammation may therefore not only improve acute SARS-CoV-2 infection outcomes, but also improve morbidity and mortality associated with subsequent multisystem inflammation.

## Author contributions

IP KB, JB, and CA designed, wrote, and edited the manuscript. All authors contributed to literature screening, writing, and editing and approved the submitted version.

## Funding

IP and CA are supported by grants from the National Institutes of Health/National Institute of Diabetes and Digestive and Kidney Diseases/National Heart, Lung and Blood Institute/National Institute of Allergy and Infectious Diseases: R01 DK130481 (IP), R01 DK113919 (IP/CA), R01 DK119936 (CA), R01 DK131476 (CA), RO1 HL117715 (IP), R01 HL123096 (IP), R01 HL154862 (IP), R01 AI119346 (CA). This study was funded, in part, by the Division of Intramural Research, NIAID. The content of this publication does not necessarily reflect the views or policies of the Department of Health and Human Services, nor does mention of trade names, commercial products, or organizations imply endorsement by the U.S. Government. The funders had no role in study design, data collection and analysis, decision to publish, or preparation of the manuscript.

## Acknowledgement

**Figure 1** was created with BioRender.com.

## Conflict of interest

The authors declare that the research was conducted in the absence of any commercial or financial relationships that could be construed as a potential conflict of interest.

## Publisher’s note

All claims expressed in this article are solely those of the authors and do not necessarily represent those of their affiliated organizations, or those of the publisher, the editors and the reviewers. Any product that may be evaluated in this article, or claim that may be made by its manufacturer, is not guaranteed or endorsed by the publisher.
